# Comparison of different genetic testing modalities applied in paediatric patients with steroid-resistant nephrotic syndrome

**DOI:** 10.1186/s13052-024-01655-4

**Published:** 2024-04-23

**Authors:** Xueting Cheng, Jiahuan Chen, Xueying Yang, Han Chan, Xia Yang, Jia Jiao, Anshuo Wang, Gaofu Zhang, Xuelan Chen, Xiaoqin Li, Mo Wang, Baohui Yang, Haiping Yang, Qiu Li

**Affiliations:** Department of Nephrology, National Clinical Research Center for Child Health and Disorders, Ministry of Education Key Laboratory of Child Development and Disorders, Chongqing Key Laboratory of Pediatrics, Children’s Hospital of Chongqing Medical University, Chongqing, China

**Keywords:** Genetic testing, Steroid-resistant nephrotic syndrome, Children

## Abstract

**Background:**

Steroid-resistant nephrotic syndrome (SRNS) are monogenic in some cases, however, there are still no clear guidelines on genetic testing in the clinical practice of SRNS in children.

**Methods:**

Three hundred thirty-two children were diagnosed with SRNS, and all children underwent genetic testing, including gene panels and/or whole-exome/genome sequencing (WES/WGS), during treatment. We analysed the relationship between clinical manifestation and genotype, and compared different genetic testing methods’ detection rates and prices.

**Results:**

In this study, 30.12% (100/332) of children diagnosed with SRNS had monogenic causes of the disease. With 33.7% (122/332) of children achieving complete remission, 88.5% (108/122) received steroids combined with tacrolimus (TAC). In detectability, WES increased by 8.69% (4/46) on gene panel testing, while WGS increased by 4.27% (5/117) on WES, and WES was approximately 1/7 of the price of WGS for every further 1% increase in pathogenicity.

**Conclusions:**

We verified that steroids combined with TAC were the most effective option in paediatric SRNS. In detection efficiency, we found that WGS was the highest, followed by WES. The panel was the lowest, but the most cost-effective method when considering the economic-benefit ratio, and thus it should be recommended first in SRNS.

**Supplementary Information:**

The online version contains supplementary material available at 10.1186/s13052-024-01655-4.

## Introduction

Nephrotic syndrome (NS) is clinically characterized by massive proteinuria, hypoproteinaemia, and/or oedema [1]. Idiopathic nephrotic syndrome (INS) accounts for approximately 90% of NS cases in the paediatric period [2, 3]. Oral glucocorticoids are the first-line treatment option for INS. Approximately 85% of patients have been found to achieve complete remission of urinary protein after adequate steroid therapy [4–6]. Some children who remain positive for urine protein after 4–6 weeks of oral glucocorticoid therapy are diagnosed with SRNS. Clinical practice guidelines recommend genetic testing for children with SRNS [7, 8]. Now, 10–30% of children with SRNS are diagnosed with monogenic SRNS [9]. Timely genetic testing can provide an unequivocal diagnosis for patients and families and reduce unnecessary damage during treatment, and may also uncover a form of SRNS that is amenable to treatment (e.g., coenzyme Q10) [10–13], which would allow us to provide individualized treatment for each patient.

Currently, the main genetic testing approaches used for children with SRNS include traditional Sanger sequencing and next-generation sequencing (NGS). Three NGS approaches for genetic diagnosis are now available in the clinical setting: (1) gene panels; (2) WES; and (3) WGS [14]. When the relationship between genes and clinical phenotypes is unclear. Genetic panel analysis is recommended for the next step, but it could have the limit of missing important disease-associated causative genes that have not been identified [15, 16]. The range of WES sequencing is the entire exome, so it is an unbiased approach that is more effective than panel sequencing [17–19]. However, WES detects only 1–2% of the whole genomic protein-coding regions. WGS can provide complete genetic testing data for individuals, but its high cost and long analytic period prevent its use in clinical applications [20, 21], while reports of the positive diagnostic rate and application value of WGS in the clinical application of SRNS have been lacking.

Genetic testing has been rapidly advancing to provide physicians and patients with a significant benefit in the understanding and treatment of disease. The currently recommended first-line immunosuppressive treatment is the use of calcineurin inhibitors (CNIs) when blood levels are available for testing. However, a proportion of patients with poor outcomes still progress to end-stage renal disease [22, 23]. We hope that, through this study, we can explore a more rational choice of genetic testing strategies in clinical practice. By analysing the correlation between the genetic and clinical phenotypes of nephrotic syndrome, we can provide a reference for the precise treatment of this disease.

## Materials and methods

### Patients

This study was approved by the Ethics Committee of the Children’s Hospital of Chongqing Medical University (No.2022 − 580). A total of 332 patients with gene sequencing for SRNS who were treated and followed up at the Children’s Hospital of Chongqing Medical University, from January 2010 to August 2022, were recruited. The information collected included general information, family history, laboratory data, renal pathology biopsy results, gene sequencing results, medication use, recurrence, and treatment progress of the paediatric patients. The inclusion criteria for this study were children with an age of onset < 18 years old and a clinical diagnosis of nephrotic syndrome or nephrotic range proteinuria. Exclusion criteria were the following: [1] clinical information about the child was not fully accessible; [2] no individual genetic testing was performed; and [3] a diagnosis of secondary nephrotic syndrome was considered.

All relative definitions were based on International Pediatric Nephrology Association guidelines and summarized in Table 1.

**Table 1:** Definitions relating to the nephrotic syndrome in children

Term	Definition
**Nephrotic-range proteinuria**	UPCR ≥ 200 mg/mmol (2 mg/mg) in a spot urine, or proteinuria ≥ 1000 mg/m2 per day in a 24 h urine sample corresponding to 3 + (300–1000 mg/ dL) or 4 + (≥ 1000 mg/dL) by urine dipstick
**Nephrotic syndrome**	Nephrotic-range proteinuria and either hypoalbuminemia (serum albumin < 30 g/L) or edema when serum albumin is not available
**Complete remission**	UPCR (based on first morning void or 24 h urine sample) ≤ 20 mg/mmol (0.2 mg/mg) or < 100 mg/m2 per day, respectively, or negative or trace dipstick on three or more consecutive days
**Partial remission**	UPCR (based on first morning void or 24 h urine sample) > 20 but < 200 mg/mmol (> 0.2 mg/mg but < 2 mg/mg) and serum albumin ≥ 30 g/L
**No remission**	No response was the presence of nephrotic range proteinuria, serum albumin of < 25 g/L, or edema
**SRNS**	Lack of complete remission within 4 weeks of treatment with PDN at standard dose
**CNI-resistant SRNS**	Absence of at least partial remission after 6 months of treatment with a CNI at adequate doses and/or levels.

UPCR, urine protein/creatinine ratio, SRNS steroid-resistant nephrotic syndrome, CNI calcineurin inhibitor

### Statistical analysis

This study used Microsoft Excel spreadsheets for data collection and IBM SPSS Statistics software, version 26.0, for data analysis. The measurement data were expressed as the number of cases (percentage) percentages, and the X2 test was used for comparison between groups. The measurement data conforming to a normal distribution were expressed as the mean ± standard deviation. Nonnormally distributed measures were expressed using the median (quartiles). Renal survival refers to the probability that the study subject did not reach the observation endpoint at the end of the follow-up. Observation endpoints refer to children on renal dialysis or peritoneal dialysis, receiving renal transplantation, or dying, with cut-off events being missed visits or deaths from nonnephrotic causes. Differences were considered statistically significant at *P* < 0.05.

### Genetic testing and renal biopsy

Genomic DNA is extracted using the QIAamp DNA Mini Kit and the appropriate DNA is screened by quality control for fragmentation. A DNA Sample Prep Reagent Set is then used to perform library preparation through a process of end repair, adapter ligation and PCR amplification. The enrichment libraries were sequenced on DNBSEQ (DNBSEQ-T7) for paired readings of 150 bp. If the patient wants to perform a co-segregation verification of the family line, genomic DNA is obtained from all available family members for Sanger sequencing. After sequencing, the raw data were saved as a FASTQ format. Bioinformatics analysis was then conducted to detect the harmfulness of the variation by correlating it with multiple databases.

According to the American College of Medical Genetics and Genomics and the Association for Clinical Genomic Science criteria, Genetic reports used specific standard terminology: ‘pathogenic’, ‘likely pathogenic’, ‘uncertain significance’, ‘likely benign’, and ‘benign’ to describe the mutations [24]. Gene variants pathogenicity was reviewed by a geneticist.

Also, according to the IPNA (International Pediatric Nephrology Association), we recommend a renal biopsy in all children diagnosed with SRNS, except in known infection or malignancy-associated secondary disease or potentially in patients with familial and/or syndromic cases or genetic causes of SRNS [8]. However, not every patient underwent this test because some children were too young to cooperate with the examination, or their guardian did not accept it for their own consideration.

### Modeled cost analysis

We performed a model cost analysis. The tests required for diagnosis were summarized according to guidelines, local routine clinical practice. Tests were categorized into 1–3 Tiers based on necessity, price, and complexity, as detailed in Supplementary Table 1 (see Additional file 1). We modeled two possible diagnostic trajectories: [1] late genetic testing model, after a full diagnostic pathway of Tiers 1, 2, and 3, finally using genetic testing; and [2] early genetic testing model, using genetic testing right after the Tier 1. The cost difference between the two models was compared.

### Real-life cost analysis

We performed a real-life retrospective cost analysis of the diagnostic pathway during the initial hospitalization, and additionally analyzed the diagnostic costs for children who underwent genetic testing twice. A total of 40 patients were randomly selected from single-gene-positive SRNS children with full diagnostic pathway information before genetic testing was performed. The real-life costs were compared with costs from the early genetic testing model.

In all cost analyses, we focused exclusively on laboratory, instrumentation, operational, and radiographic costs incurred during hospitalization, with each expense calculated according to local policies and prices denominated in US dollars.

## Results

### Patient characteristics

We enrolled 332 SRNS patients in our single-center study, including 219 boys and 113 girls (males: females 1:0.52, *P* = 0.044 < 0.05, with significant differences), with an average age of 69.9 ± 50.4 months old, who had used at least one type of genetic test. The baseline characteristics of all SRNS patients are summarized in Table 2. Supplementary Table 1 (see Additional file 1) lists the results of all detected causative mutations.

Table 2. Baseline characteristics of the study population.

**Table 2:** Baseline characteristics of the study population

Characteristics	ALL (*n* = 332)	Monogenic Disease (*n* = 100)	Genetic-Testing Negative(*n* = 232)
**Gender(male/female)**	219/113	58/42	161/71
**Age at onset(months), median (IQR)**	69.9 ± 50.4	68.2 ± 53.6	70.6 ± 49.1
**Clinical classification**			
** Without glomerular hematuria, renal insufficiency and hypocomplementemia**, ***n*(%)**	143/332(43.1)	30/100(30.0)	113/232(48.7)
** With glomerular hematuria or/and renal insufficiency or/and hypocomplementemia**, ***n*(%)**	189/332(56.9)	70/100(70.0)	119/232(51.3)
**Histopathological findings**			
**MCD**, ***n*(%)**	85/168(50.6)	13/42(30.9)	72/126(57.1)
**FSGS**, ***n*(%)**	55/168(32.7)	18/42(42.9)	37/126(29.4)
**MSPGN**, ***n*(%)**	7/168(4.2)	4/42(9.5)	3/126(2.4)
**MPGN**, ***n*(%)**	5/168(3.0)	1/42(2.4)	4/126(3.2)
**Others**^**1**^, ***n*(%)**	16/168(9.5)	6/42(14.3)	10/126 (7.9)
**None**, ***n*(%)**	164/332(49.4)	58/100(58.0)	106/232(45.7)
**Length of follow-up(months), median (IQR)**	25.4(10.0-47.6)	23.5(3.1–44.1)	25.8(11.6–48.8)
**Renal survival rate**, ***n*(%)**	308/332(92.8)	80/100(80.0)	228/232(98.3)
**Treatment**			
**Steroid + TAC**, ***n*(%)**	214/332(64.4)	52/100(52.0)	162/232(69.8)
**Complete**, ***n*(%)**	108/214(50.5)	15/52(28.8)	93/162(57.4)
**Particle**, ***n*(%)**	52/214(24.3)	9/52(17.3)	43/162(26.5)
**Resistance**, ***n*(%)**	50/214(23.3)	25/52(48.1)	25/162(15.4)
**Unknow**, ***n*(%)**	4/214(1.9)	3/52(5.8)	1/162(0.6)
**Steroid + MMF/CTX**, ***n*(%)**	53/332(16.0)	13/100(13.0)	40/232(17.2)
**Complete**, ***n*(%)**	12/53(22.6)	2/13(15.4)	10/40 [25]
**Particle**, ***n*(%)**	13/53(24.5)	1/13(7.7)	12/40 [30]
**Resistance**, ***n*(%)**	24/53(45.3)	10/13(76.9)	14/40 [35]
**Unknow**, ***n*(%)**	4/53(7.6)	0/13(0)	4/40 [10]
**Others**^**2**^, ***n*(%)**	65/332(19.6)	35/100(35.0)	30/232(12.9)
**Complete**, ***n*(%)**	2/65(3.1)	0/35(0)	2/30(6.7)
**Particle**, ***n*(%)**	1/65(1.5)	0/35(0)	1/30(3.3)
**Resistance**, ***n*(%)**	49/65(75.4)	33/35(94.3)	16/30(53.3)
**Unknow**, ***n*(%)**	13/65 [20]	2/35(5.7)	11/30(36.7)

Others1: Alport syndrome, various glomerulonephritis, Membranous nephropathy

Others2: Patients treated with steroid but not with TAC, CTX, MMF

Unknow: Less than 6 months of follow-up or unable to determine remission due to infrequent follow-ups within 6 months

### General information

**Fig. 1:**
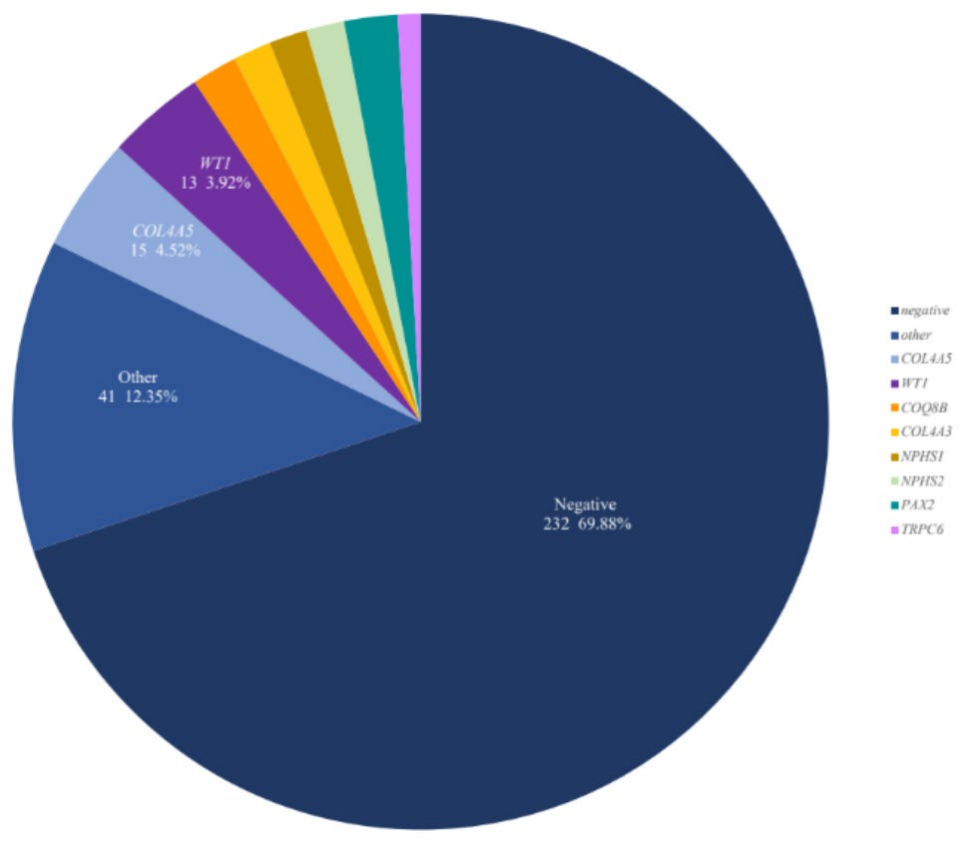
The proportion of gene distribution in SRNS patients. Among them, COL4A5 accounted for the most, 4.52% (15/332), followed by WT1 at 3.92% (13/332), PAX2 at 2.10% (7/332), COQ8B at 1.80% (6/332), COL4A3, NPHS1, and NPHS2 all at 1.51% (5/332), TRPC6 0.90% (3/332); a total of 41 other genes, accounting for 12.35% (41/332)

We performed different sequencing in a single cohort of 332 SRNS children, and causative mutations detected by any method were counted as positive. A total of 100 SRNS-related causative mutations were detected, counting the positive rate of single-gene was 30.12% (100/332). The family history significantly increased the diagnostic rate compared to children without it (15.79% versus 4.61%, *P* = 0.01 < 0.05). Forty types of causative genes related to SRNS disease were found. The distribution of these genes is shown in Fig. 1, of which *COL4A5* was the most detected, with a total of 15, followed by *WT1*, with a total of 13.

### Clinical manifestations with causative mutations

SRNS patients with causative mutations included 58 boys and 42 girls (males: females 1:0.72). The onset of illness ranged from 1 month to 15.2 years of age, and the median age in individuals in whom a causative mutation was detected was 68.2 ± 53.6 months old.

**Fig. 2:**
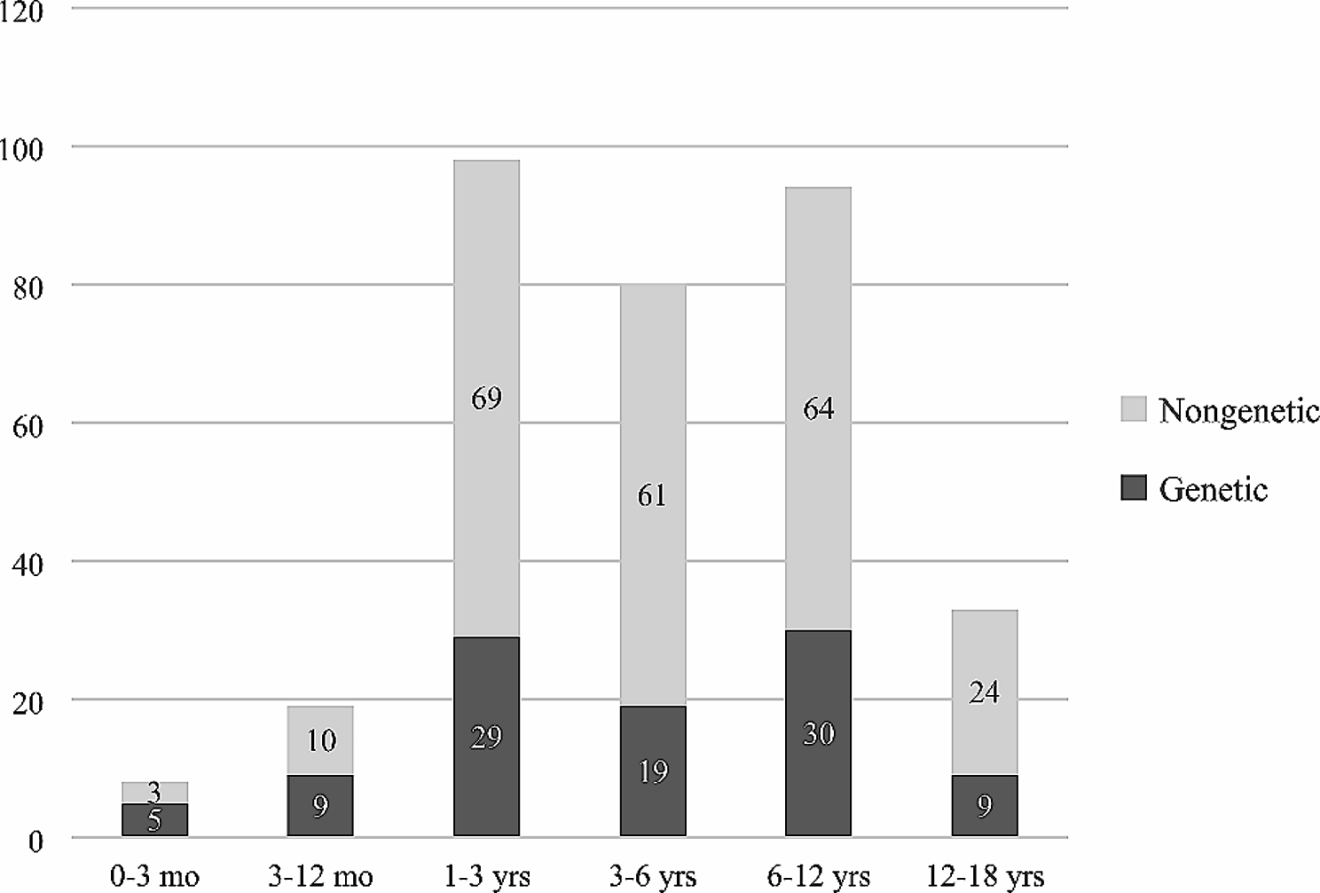
Age of onset distribution (in years) for 332 SRNS patients. Numbers on bars represent the proportion of affected individuals in different age groups; Dark: patients with a causative mutation detected; Gray: patients without a causative mutation

**Fig. 3:**
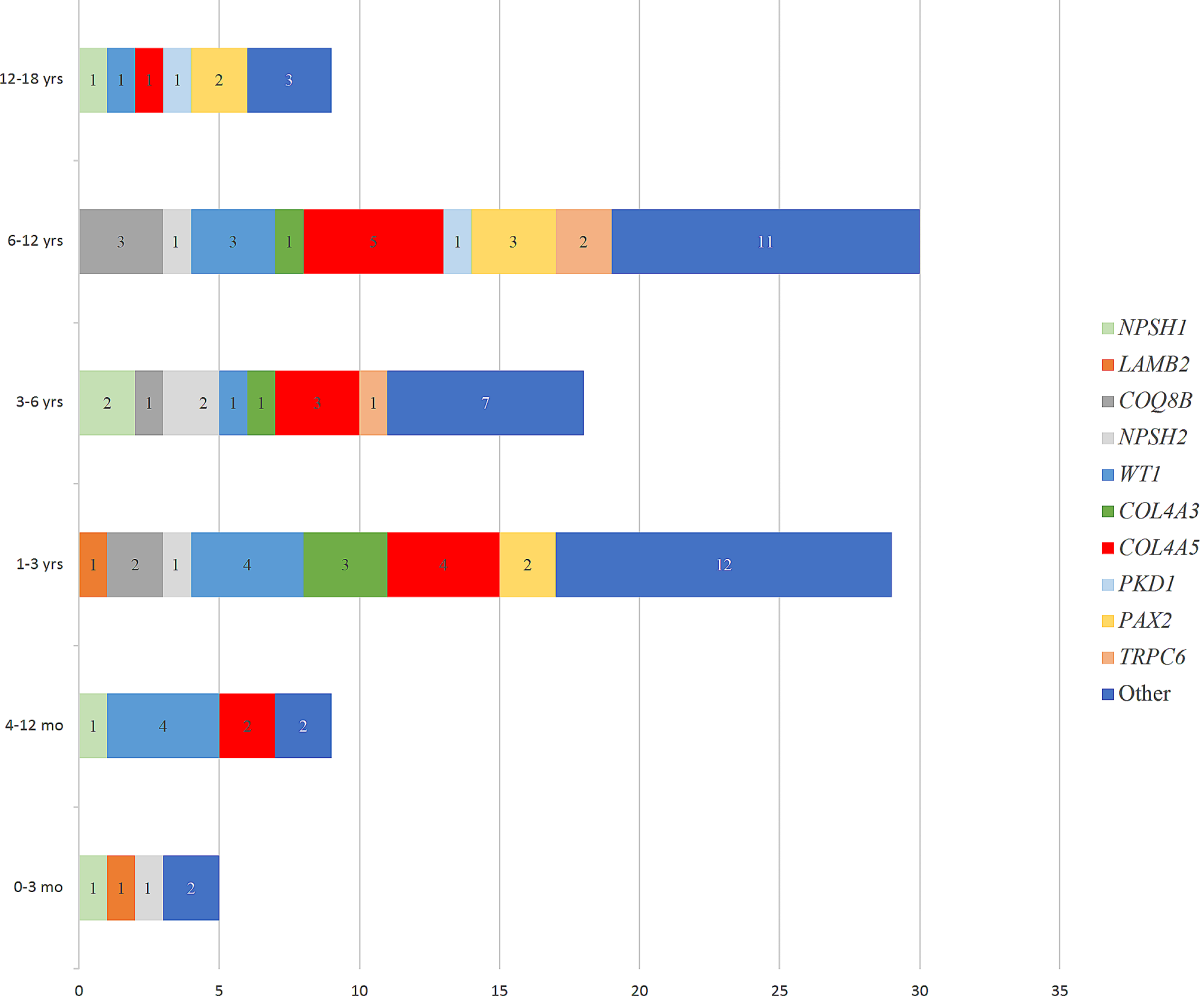
Number of patients with causative mutation detected per gene per age group. Numbers on bars represent proportion of causative mutations in different age group: onset in the first 3 months of life (5 patients); 3–12 months (9 patients); aged 1–3 years (29 patients); aged 3–6 years (18 patients); aged 6–12 years (30 patients); and aged 12–18 years (9 patients)

The proportions of patients with detected causative mutations were as follows: onset in the first 3 months of life (5/8, 62.5%); 3–12 months (9/19, 47.4%); aged 1–3 years (29/98, 29.6%); aged 3–6 years (18/80, 22.5%); aged 6–12 years (30/94, 31.9%); and aged 12–18 years (9/33, 27.3%). *WT1* was more common in patients aged 3 months to 3 years old, and *COL4A5* in children aged 1 to 12 years old. The results for the detection of causative mutations at different ages are shown in Fig. 2, and the specific distribution of these causative genes is shown in Fig. 3.

### Clinical phenotype and renal biopsy

Among children with causative mutations, 70.0% (70 of 100) of SRNS patients had glomerular haematuria and/or renal insufficiency and/or hypocomplementaemia at onset, and 30 children (30.0%) did not have the above symptoms.

**Fig. 4:**
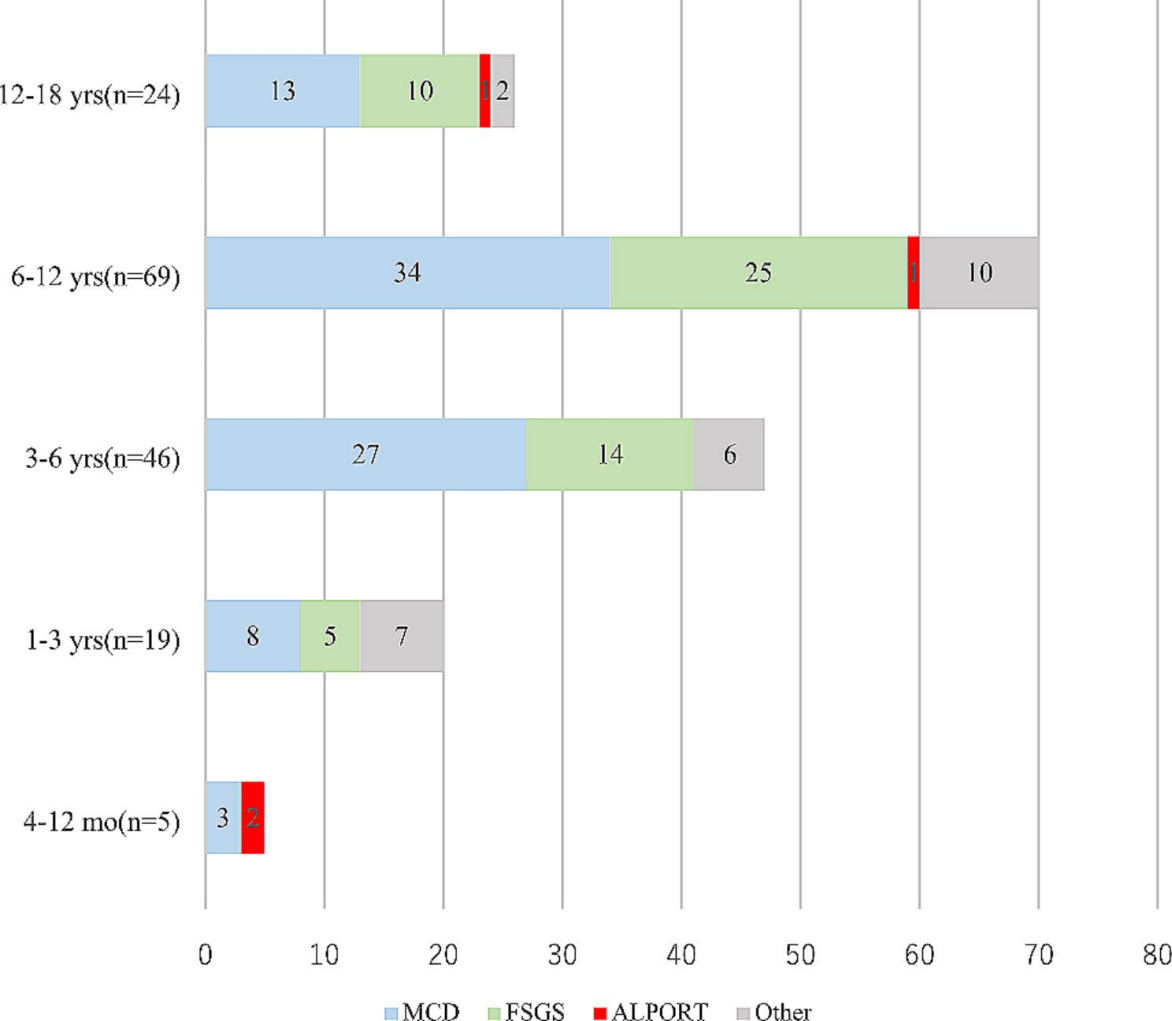
SRNS pathological types of different age groups. Other: Alport syndrome, various glomerulonephritis, membranous nephropathy

**Fig. 5:**
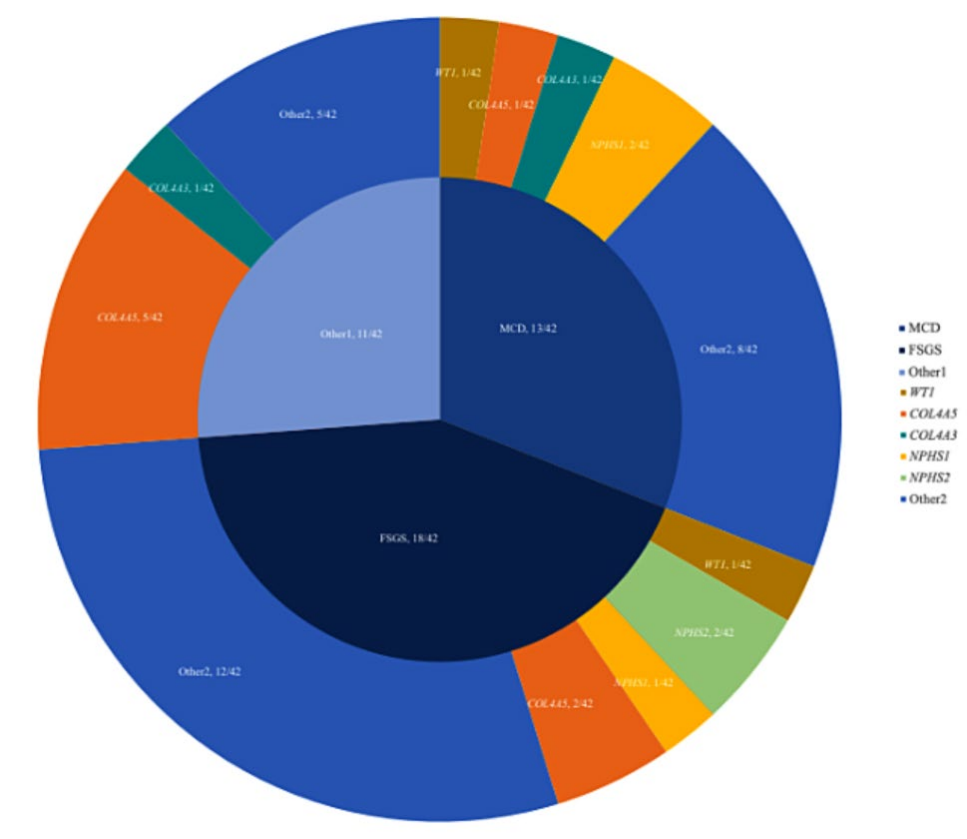
The distribution of different causative genes in different renal biopsy types. A total of 42 children were detected with causative mutations and completed renal biopsies. Inner segments represent the numbers and fractions of renal biopsy types, specifically as follows: MCD, 13 out of 42; FSGS, 18 out of 42; Other1, 11 out of 42. Outer segments represent for each renal biopsy group the relative fraction of different causative mutations. We described the 5 specific causative mutations that accounted for a large share or occurred frequently, others are grouped in other2. The distribution is as follows: WT1 (1 out of 42), COL4A3 (1 out of 42), COL4A5 (1 out of 42), NPHS1 (2 out of 42) and other causative mutations (8 out of 42) were detected in MCD group. WT1 (1 out of 42), COL4A5 (2 out of 42), NPHS1 (1 out of 42), NPHS2 (2 out of 42) and other causative mutations (12 out of 42) were detected in FSGS group. COL4A3 (1 out of 42), COL4A5 (5 out of 42) and other causative mutations (5 out of 42) were detected in other renal pathological group. Other1: Alport syndrome, various glomerulonephritis, Membranous nephropathy. Other2: Other causative genes detected in patients with different pathological groups

A total of 168 of 332 SRNS patients underwent renal biopsy at different ages but were not chosen by the age of 0–3 months old. The pathological phenotypes in each age group are shown in Fig. 4. Pathological changes were seen to be predominantly minimal change disease (MCD) and focal segmental glomerulosclerosis (FSGS), with MCD being higher than FSGS. A renal biopsy was performed in 42 of 100 individuals with disease-causing mutations, showing 13 patients with MCD, 18 patients with FSGS, 4 patients with mesangial proliferative glomerulonephritis (MsPGN), 1 patient with membrano-proliferative glomerulonephritis (MPGN), and 6 patients with other conditions. The distribution of different causative genes in different renal biopsy types is shown in Fig. 5.

### Therapy

In this study, a total of 332 children were included, and 122 (36.7%) achieved complete remission of urinary protein, including 17 (5.1%) children who were monogenic positive and 105 (31.6%) with negative genetic testing. Partial remission of urinary protein occurred in 66 (19.9%) paediatric patients, of whom 10 (3.0%) were monogenic positive and 56 (16.9%) were genetic testing negative. During treatment, 123 children developed immunosuppression tolerance and did not meet the criteria for proteinuria remission, including 68 (20.5%) children who were monogenic positive and 55 (16.6%) children who were negative by genetic testing negative.

There were different options for treatment, as shown in Table 2. A total of 122 children achieved complete remission of urinary protein, 108 children chose steroids + TAC, 12 children chose steroids + mycophenolate mofetil (MMF)\cyclophosphamide (CTX), and 2 children did not choose immunosuppressive therapy. Among the TAC group, 15 were monogenic positive, and 93 were negative. Of the 12 MMF\CTX group children, only 2 were monogenic positive. In children with other non-immunosuppression therapies, 2 achieved complete remission and were both genetically negative, and most children did not achieve remission during the process of treatment.

### Comparison of genetic testing modalities and costs

#### Process of acquisition

**Fig. 6:**
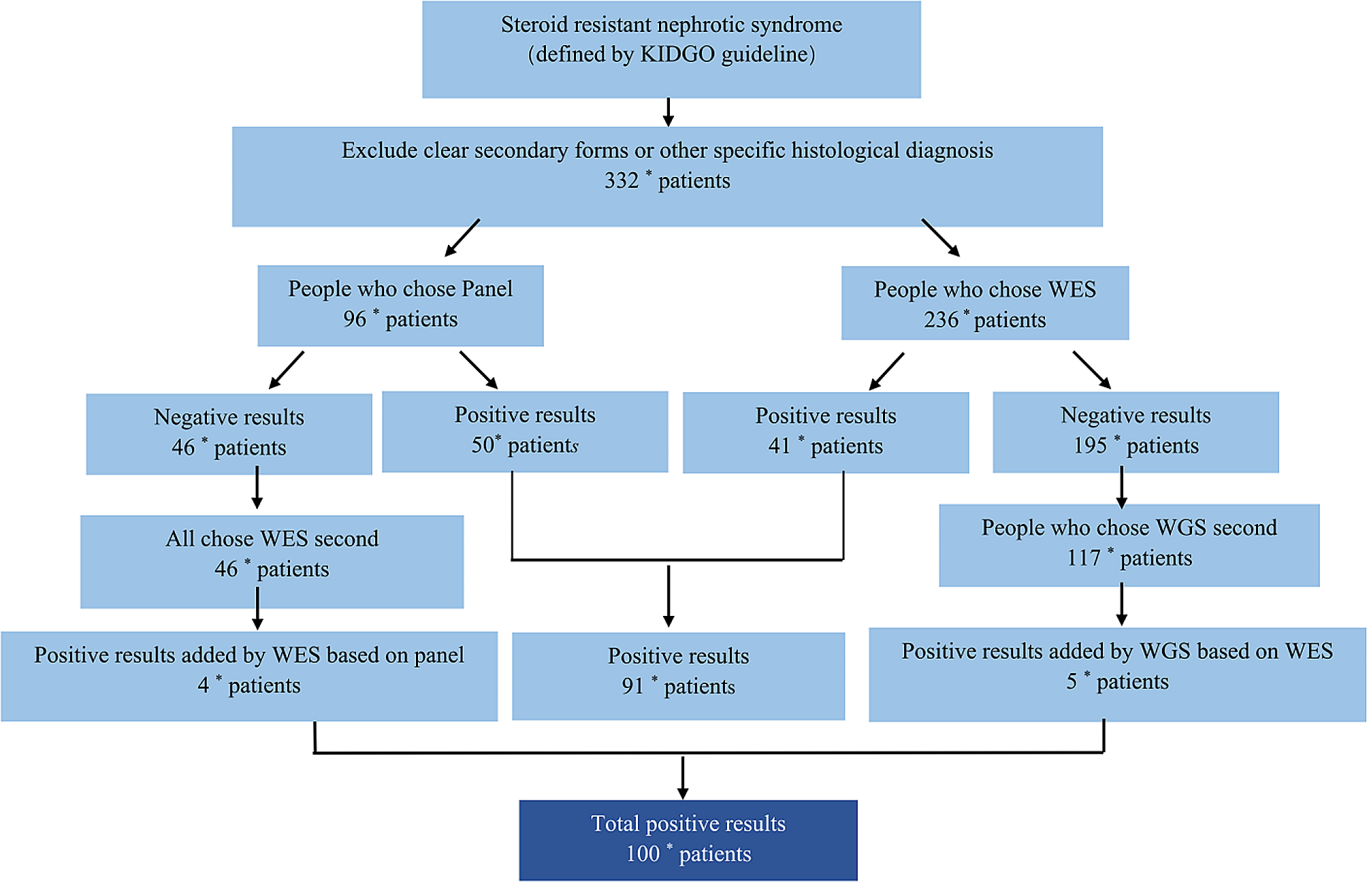
Flowchart for the selection of genetic testing strategies for SRNS patients. The first strategy was used in children who chose the panel first with 96 in total, 50 of whom had causative mutations, and 46 had negative results; all of the 46 panel-negative children chose to have further WES tests, and 4 additional causative mutations were detected in the end. Another group included 236 children who chose WES first, 41 of whom had causative mutations, and 195 had negative results. Of the 195 WES-negative children, 117 chose to be further tested for WGS, and finally, 5 additional causative mutations were detected

In our study, the choice of genetic tests can be divided into two different strategies. The details of the testing process are shown in Fig. 6. In the first strategy, all 46 panel-negative patients chose further WES, and 4 additional mutations were found, increasing the positive rate by 8.69% (4/46). Conversely, 117 of 195 WES-negative patients chose further WGS, and 5 additional mutations were found, increasing the positive rate by 4.27% (5/117).

### Analysis of additional detected genes

When analysing the results obtained by the first-selected panel or WES when the second genetic tests were not yet performed, we found that the overall causative mutation detection rates were 52.08% (50/96) and 17.37% (41/236), respectively. The types of causative genes were also analysed at the same time, and we found that a total of 19 types were detected by gene panel testing and 26 types were detected by WES. The top 3 causative mutations detected by the panel were *COL4A5* (13 cases), *WT1* (9 cases) and *PAX2* (6 cases), accounting for 56.0%. The top mutation detected by WES was *COQ8B* (5 cases), followed by *WT1* (3 cases) and *NPHS2* (3 cases); all three genes accounted for 26.83%.

WES detected 4 additional causative mutations based on the negative panel outcome: *TTC21B*, *COL4A5*, *WT1*, and *COQ8B*. *WT1* and *COQ8B* were detected in children whose first-selected mitochondria panel did not contain those two genes. Among the remaining 2 causative mutations, *TTC21B* detected 2 point mutations in the first panel, but subsequent Sanger sequencing found that both of the mutations were from this child’s mother, who did not show any phenotype of NS. However, later WES considered the incomplete penetrance and again concluded that it was a causative mutation. The *COL4A5* mutation type was exon deletion. After asking the gene company, we found that the panel was not very good at detecting exon deletions in this case due to insufficient probe density at the time.

Five additional causative mutations were detected by WGS based on negative WES, including four species, which were *NIPBL*, *COL4A3*, *TRNL1*, and *COL4A4*. Two *TRNL1* mutations were located in mitochondria. The remaining 3 causative mutations were all missense mutations. The *COL4A3* mutation was not found in the first WES but was found with WGS; however, we later decided to reanalyse the WES data and finally reached a positive result for *COL4A3*, similar to the WGS results. Generally, WES will not miss the *COL4A4* mutation. In the case of *COL4A4*, we consulted the genomic company about this matter and learned that the patient was sequenced at the first genomic company, then copied the sequencing date results to the second genomic company and went WES pure data analysis. WES failed to find the mutation for the first time probably due to poor sequencing quality at the first company, but later WGS detected the *COL4A4* mutation at the second genomic company successfully. The last *NIPBL* mutation was initially identified by WES, but it was not considered a causative mutation. Subsequent WGS added parental verification and eventually designated it as a spontaneously mutated disease-causing gene.

### Economic-benefit ratio

The Children’s Hospital of Chongqing Medical University is the National Clinical Research Center for Child Health and Disorders, located in southwest China. A comprehensive look at the quotes for genetic tests from two genetic companies commonly found in this region showed that a single renal disease panel cost approximately $253, a single WES cost approximately $393, and a single WGS cost approximately $562. If Trios were chosen for family line verification, the price was higher: up to $815 for Trios WES and $1,405 for Trios WGS.

For genetic testing benefits when first genetically tested, the first-chosen panel positive rate was 52.08% (50/96) and cost approximately $466 for every 1%, and the first-chosen WES positive rate was 17.37% (41/236) and cost approximately $5,339 for every 1%. All 46 panel-negative individuals selected WES to continue testing, and an 8.69% (4/46) additional positive rate was generated by the additional selection of WES with a total additional cost of $18,078 or $2,080 for every 1% boost; 4.27% (5/117) was generated by further WGS based on WES-negative children with a total additional cost of $65,754 or $15,399 for every 1%. In comparison, WES costs approximately 1/7th the price of WGS for every 1% increase in pathogenicity detection.

### Model analysis and cost comparison

**Fig. 7:**
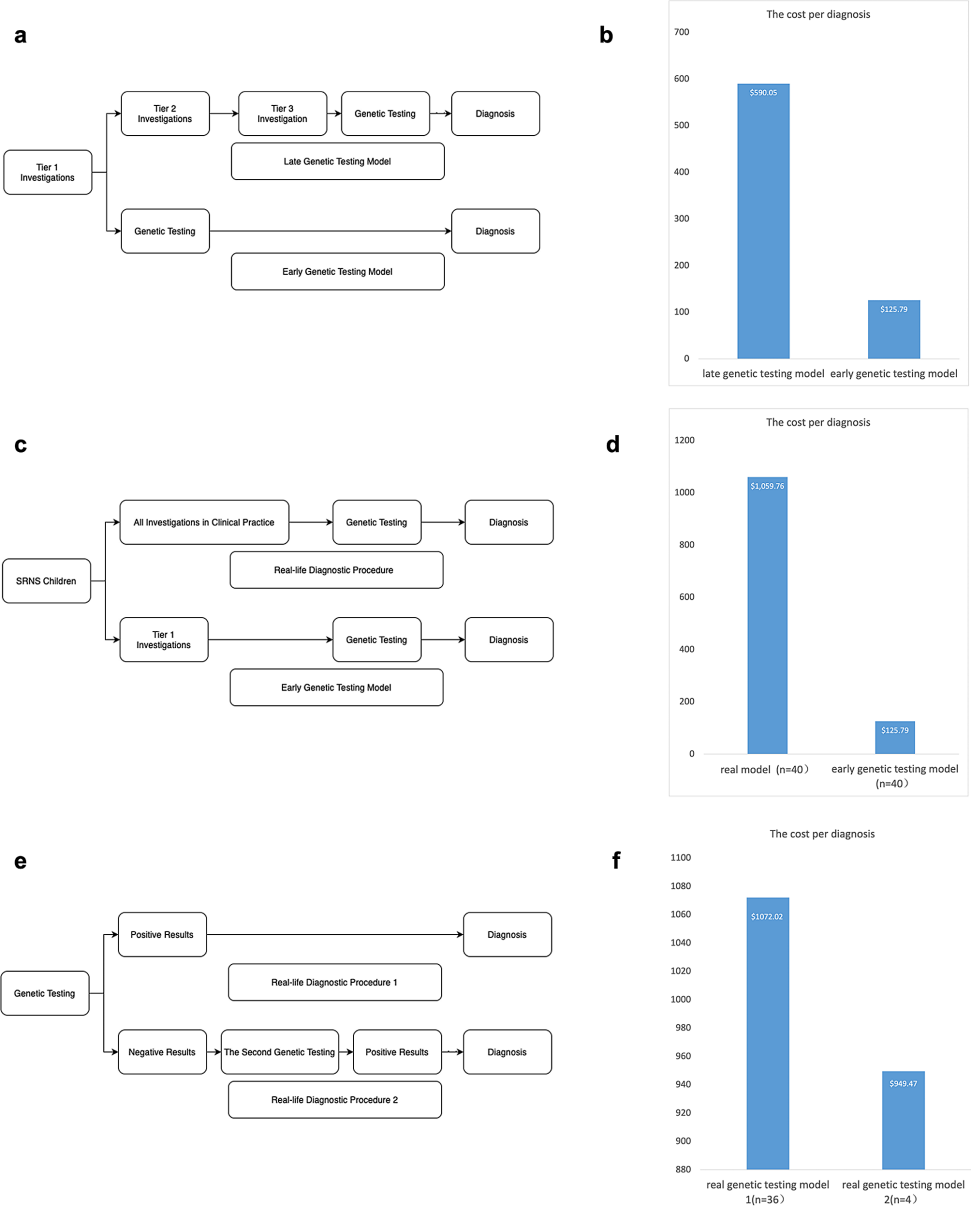
Cost analysis. (**a**) Modeling diagnostic trajectories for SRNS patients with suspected single gene mutations. tier 1 includes baseline investigations, tier 2 and tier 3 include increasingly complex and/or costly investigations. Diagnostic tests are based on current guidelines and local clinical practice. In the late genetic testing model, patients first undergo Tier 1–3 investigations, followed by genetic testing. In the early genetic testing model, patients go through only Tier 1 and then directly to genetic testing. If positive results were not obtained from the test, further genetic testing would be performed until a final diagnosis was reached. In both models, the prices for all checks were the same as the actual local prices. (**b**) Comparison of the average cost per diagnosis for late versus early genetic testing models in the study population. (**c**) Comparison of real-life diagnostic procedure costs with early genetic testing model. Retrieve all costs incurred by the real-life diagnostic procedure for 40 children. Compare the average cost incurred by the real-life diagnostic pathway to the cost of the early genetic testing model. (**d**) Average real-life versus early genetic testing model cost. (**e**) The real genetic test model 1 was the cost of the first genetic test to determine the single gene cause in the 40 patients described above, for a total of 36 children diagnosed with the first genetic test. The real genetic test model 2 was the cost of the remaining 4 children who had a second genetic test to determine a single gene cause. (**f**) Comparison of the actual costs of the real genetic testing model 1 with the real genetic testing model 2

We compared the two different models along with the actual cost analysis model and showed them in Fig. 7. Figure 7b shows that the early genetic testing model has reduced the cost per diagnosis of the late genetic testing model by 79% ($464); Fig. 7d shows that the early genetic testing model is 88% ($934) lower than the real-life cost of the 40-patient diagnostic pathway; Fig. 7f shows that the actual cost of a twice genetic test is about 89% ($949) more to diagnose than only once. These results suggested that early and appropriately selected genetic testing can save the cost of diagnosing single-gene-positive SRNS.

## Discussion

In this study, we found 30.12% causative mutations in Chinese children with SRNS. Differently from previous studies, we included three types of genetic testing and performed different detections in all children, each undergoing at least one. This process enabled us to analyse the clinical phenotype and genotype relationship, compare the detection differences between different testing modalities, and calculate the costs and benefits.

Previous studies have reported that detecting causative mutations is inversely related to the age of disease onset [15]. We also found that detection varied by age and showed a downward trend. The highest proportion of 62.5% was found in those 0–3 months old, which is almost equal to the 69.4% reported in other studies that included children with single gene-positive NS in 526 families [25]. The majority of single-gene-positive SRNS patients exhibited glomerular haematuria and/or renal insufficiency and/or hypocomplementaemia at the beginning of this disease, and the most common histopathologies were FSGS (42.9%) and MCD (31.0%). These results remind us that genetic testing results could allow more individualized management and follow-up, as well as more precise prognostic evaluation and genetic counseling to families, in addition to the eventual potentiality of avoiding invasive investigations.

Regarding treatment, 80.7% of SRNS received different immunosuppressants combined with steroids. A total of 52.0% received TAC, 10.7% received MMF or CTX pulse therapy in children with monogenic mutations, 69.8% received TAC, and 17.2% received MMF or CTX pulse therapy in children without mutations. We found that the TAC group showed the highest complete remission in children both with and without mutations, especially for nonmonogenic SRNS, doubling the complete remission rate compared with the MMF\CTX group and increasing it by approximately 11% overall. In addition, we found that, in single-gene-positive children, MMF\CTX showed the highest resistance of 79.9%, while TAC showed 48.1%. Thus, early identification of children with monogenetic causative mutations could facilitate precise treatment and reduce unnecessary use of immunosuppressants.

Overall, SRNS patients with causative mutations respond poorly to steroids combined with tacrolimus, similar to previous studies [26–28]. Although genetic testing has great significance, it also has pricey economic limitations and has not been included in China’s health care security. we also require financial analyses for cost-effectiveness to perform genetic testing in children with SRNS.

Although Sanger is seen as the gold standard in this area, clinical phenotyping combined with panel analysis is the most cost-effective approach for mutational screening in SRNS, costing only 5-8.3% of what a traditional Sanger sequence would cost [15, 28]. Moreover, we understand that from 2017 to 2022, the number of genes included in SRNS-related panel analysis in Chongqing has more than tripled with technological advances and discovery of new genes, in addition to being cheaper now, and the time to obtain results decresed in the meantime.

However, in our study, when only one type of genetic testing was selected at the beginning, the panel showed a positive rate of 52.08%, higher than the 21–34% reported in other studies, while WES showed a positive rate of 17.37% [29]. From a clinical decision-making point of view in practical application, we recommend that children with more typical clinical symptoms/family histories/specific pathology types/poor treatment outcomes choose the more economical panel analysis first. This choice shows much pertinence, and also could explain the higher rate of panel detection.

WES can improve diagnostic yield over panels, and WGS can improve diagnostic yield over WES. Panel can lose causative mutations. This may be dependent on depth and features of the genetics laboratory. WGS can detect mitochondrial mutations while WES cannot, and reanalysis could rediscover positive results from the original WES-negative results [30].

Early studies have shown that WES could miss copy number variants (CNVs), moderately sized deletions/duplications, retro train positions, and deep intronic splice variants [31]. Microarray-based comparative genomic hybridization (array-CGH) allows a Homologous Recombination evaluation of whole genome CNVs and identifyies unbalanced chromosomal anomalies, avoiding some pathogenic variants missed by WES [32]. However, WGS includes 99% of the genome, which is noncoding and highly variable and shows more application value in reanalysis [15, 33]. WGS is now used in various disciplines and has demonstrated its clinical value, however, it is not perfect, the pathogenicity of intronic or regulatory variants remains unclear, thus leading to a type of “data waste” in WGS, furthermore, it is still the most expensive method in clinical practice [34–36].

Early and appropriate selection of genetic testing facilitates diagnostic cost savings and also avoids unnecessary follow-up therapeutic interventions, similar to other study [37–40]. It is worth noting that our actual cost analysis calculates the cost at the first hospitalization. Considering the possibility of over-calculating the amount spent on repeated tests due to changes in disease, the cost savings may have been overestimated in the end. However, our study did not account for the cost of medications for patients and may have overlooked the potential savings in steroids and various unnecessary and expensive immunosuppressants if genetic diagnosis had been performed early.

**Fig. 8:**
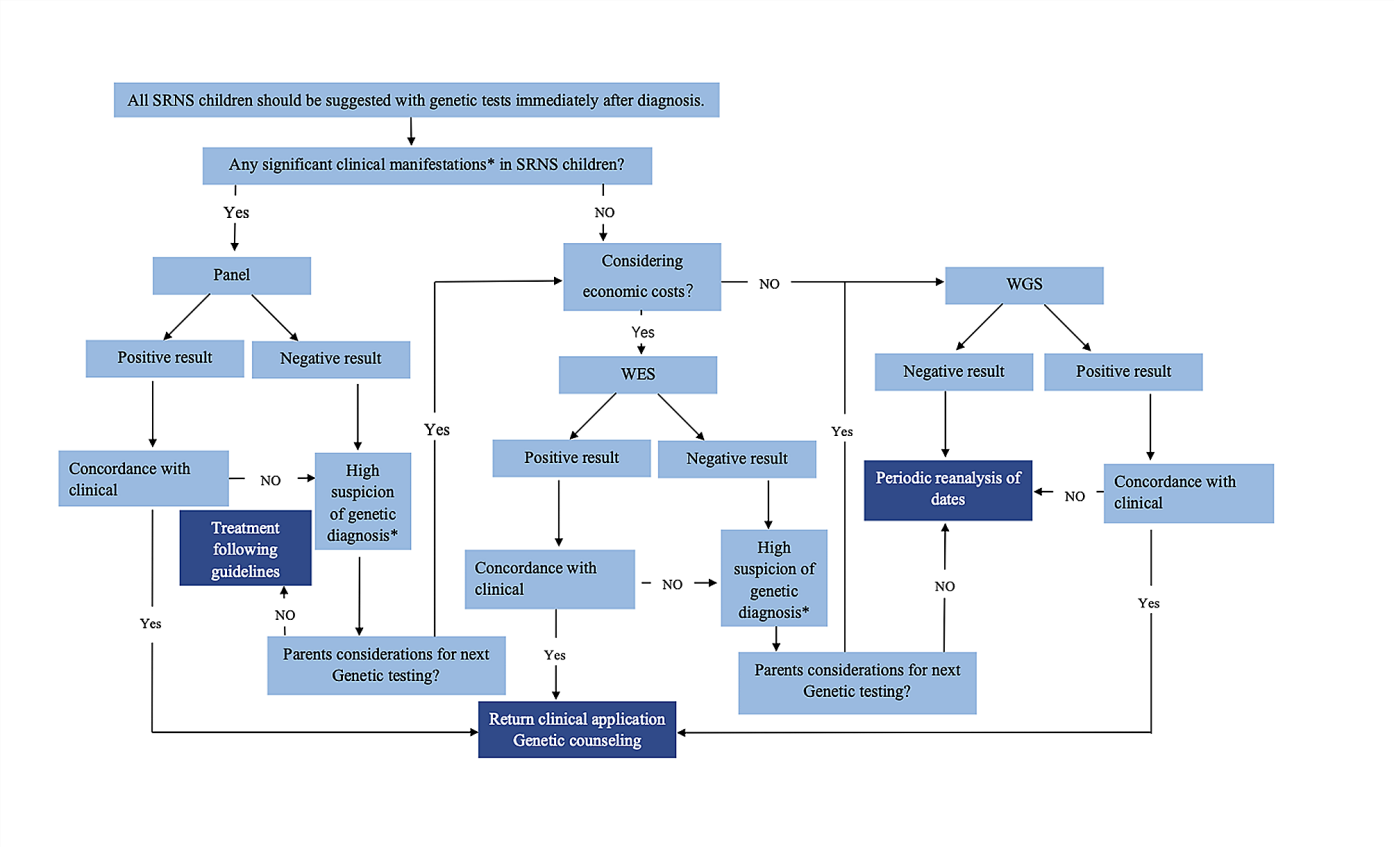
Flow chart of suggested genetic testing strategy. Significant clinical manifestations*: Positive family history, early-onset ages, abnormal renal biopsy, special extra-renal manifestations, combined with glomerular hematuria or/and renal insufficiency or/and hypocomplementemia. High suspicion of genetic diagnosis*: SRNS children with poor treatment effect or/and rapid deterioration in a short time or/and other clinical features our article did not mention

We recommend that all children with SRNS undergo genetic testing early, and the detailed approach is shown in Fig. 8. Considering the economic-benefit ratio, we suggest panel analysis first. For panel-negative patients, considering 1/7th of the price that WGS costs for every 1% increase in pathogenicity over WES and to economize, we suggest WES second. If financial considerations are not a concern, for higher diagnostic rates, it is recommended that WGS should be the first choice in panel-negative children for a “one-stop” approach, as well to avoid omission of variants of uncertain significance(VUS) and/or incidental findings, which may be reported as “Likely Pathogenic” if knowledge has changed in future.

The high cost of gene testing cannot be ignored. In the USA and the UK, genetic testing is only partly paid for by the Centers for Medicare & Medicaid Services and National Health Service, respectively [31, 41]. For developing countries, genetic testing faces inadequate financial resources and human resources, low income, and no access, making it unacquirable for many families [42–44]. There are no clear guidelines detailing the clinical applications, financial costs, and benefits of mutation screening in children with SRNS, and large, prospective studies of the clinical value of genetic testing are needed, not only to provide information for early diagnosis, precision medicine, or genetic counseling for clinical practice but also to inform coverage policies that fit the local situation.

## Conclusions

In conclusion, SRNS patients with causative mutations respond poorly and differently to steroids combined with immunosuppressive. Genetic testing contributes to the potential savings of steroids, unnecessary and costly immunosuppressants, and providing information on early diagnosis and genetic counselling. We recommend that all children with SRNS undergo genetic testing early, panel should be recommended first as the most cost-effective method. We compare the diagnostic yield in different testing modalities and the disparities in the resulting cost-benefit, wish to provide information for medical policies that fit the local situation.

### Electronic supplementary material

Below is the link to the electronic supplementary material.

SupplementaryMaterials:Table 1. List of the investigations required for the diagnosis of SRNS. Table 2. Genes to be included in next-generation sequencing for children with SRNS in our study.

## Data Availability

The datasets supporting the conclusions of this article are included within the article and its additional files.

## Abbreviations

SRNS: steroid-resistant nephrotic syndrome
WES: whole-exome sequencing
WGS: whole-genome sequencing
TAC: tacrolimus
NS: nephrotic syndrome
INS: Idiopathic nephrotic syndrome
NGS: next-generation sequencing
CNIs: calcineurin inhibitors
MCD: minimal change disease
FSGS: focal segmental glomerulosclerosis
MsPGN: mesangial proliferative glomerulonephritis
MPGN: membrano-proliferative glomerulonephritis
MMF: mycophenolate mofetil
CTX: cyclophosphamide
CNVs: copy number variants
