# Supplementary material for: Comparison of different genetic testing modalities applied in paediatric patients with steroid-resistant nephrotic syndrome
Source: Ital J Pediatr. 2024 Apr 23;50:85. doi: 10.1186/s13052-024-01655-4 (PMC11040871; doi:10.1186/s13052-024-01655-4)
Supplement: Supplementary file 1 — SupplementaryMaterials:Table 1. List of the investigations required for the diagnosis of SRNS. Table 2. Genes to be included in next-generation sequencing for children with SRNS in our study. [file 13052_2024_1655_MOESM1_ESM.docx]

Supplementary Table 1. List of the investigations required for the diagnosis of SRNS.

| Tier 1  investigations | Tier 1  costs | Tier 2  investigations | Tier 2  costs | Tier 3  investigations | Tier 3  costs | Total costs (Tier 1-2-3) |
| --- | --- | --- | --- | --- | --- | --- |
| Kidney function | 2.63＄ | ANCA | 2.77＄ | Kidney biopsy | 209.89＄ | 590.05＄ |
| Electrolytes | 8.96＄ | Immunoglobulins | 81＄ |  |  |  |
| ABG | 7.7＄ | Hepatitis B and C serology | 11.33＄ |  |  |  |
| Liver enzymes | 3.15＄ | HIV | 4.62＄ |  |  |  |
| LDH | 1.05＄ | CRP | 2.8＄ |  |  |  |
| CK-MB | 5.81＄ | ESR | 0.49＄ |  |  |  |
| Uric acid | 1.33＄ | anti-dsDNA | 3.85＄ |  |  |  |
| Glucose | 0.77＄ | Anti-GMB | 5.95＄ |  |  |  |
| Lipid profile | 4.01＄ | Anti-PLA2R | 67.15＄ |  |  |  |
| Coagulation tests | 17.2＄ | Rheumatoid Factor | 5.24＄ |  |  |  |
| Albumin | 0.46＄ | β2-miciroglobulin | 3.71＄ |  |  |  |
| Total protein | 0.46＄ | Urinary FLC | 6.72＄ |  |  |  |
| Urinalysis | 4.06＄ | ANA | 3.08＄ |  |  |  |
| Urine culture | 12.46 | ACR | 2.01＄ |  |  |  |
| Urinary p/c ratio  (or 24h proteinuria) | 2.3＄ | PCT | 34.41＄ |  |  |  |
| Abdominal ultrasound | 18.47＄ | α1-microglobulin | 4.62＄ |  |  |  |
| CBC | 1.96＄ | RBP | 3.08＄ |  |  |  |
| Parent’s urinalysis | 8.12＄ | NAG | 11.54＄ |  |  |  |
| Urinary ultrasound | 18.17＄ |  |  |  |  |  |
| Complement | 6.72＄ |  |  |  |  |  |
|  | Tot:  125.79＄ |  | Tot:  254.37＄ |  |  |  |

*We report the genomic and non-genomic investigations required for diagnosis according to current literature and local clinical practice at different tiers. Prices are reported in Dollars.*Supplementary Table 2. Genes to be included in next-generation sequencing for children with SRNS in our study.

| **Gene** | **Chromosome** | **Proband** | **Inheritance** | **Disease** | **Number of case** |
| --- | --- | --- | --- | --- | --- |
| ***ACTN4*** | 19 | het | AD | Familial and sporadic SRNS (usually adult ) | 2 |
| ***ADCK4*** | 19 | hom | AR | SRNS | 1 |
| ***ANKS6*** | 9 | het | AR | Nephronophthisis 16 | 1 |
| ***ATP7B*** | 13 | het | AR | Wilson disease | 1 |
| ***CLCN5*** | X | het | XR | Dent’s disease ± FSGS ± hypercalciuria and nephrolithiasis | 1 |
| ***COL4A3*** | 2 | het | AD | Alport’s disease/FSGS | 5 |
| ***COL4A4*** | 2 | het | AD;AR | Alport’s disease/FSGS | 2 |
| ***COL4A5*** | X | het;hemi | XD | Alport’s disease/FSGS | 15 |
| ***COQ8B*** | 19 | hom;het | AR | Nephrotic syndrome, type 9 | 6 |
| ***CRB2*** | 9 | het | AR | SRNS | 3 |
| ***EHHADH*** | 3 | het | AD | ?Fanconi renotubular syndrome 3 | 1 |
| ***FAH*** | 15 | het | AR | Tyrosinemia, type I | 1 |
| ***FGA*** | 4 | het | AD | Amyloidosis, familial visceral | 1 |
| ***GREB1L*** | 18 | het | AD | Renal hypodysplasia/aplasia 3 | 1 |
| ***IFIH1*** | 2 | het | AD | Aicardi-Goutieres syndrome 7 | 1 |
| ***INF2*** | 14 | het | AD | Familial and sporadic SRNS, FSGS-associated Charcot-Marie-Tooth neuropathy | 2 |
| ***KCNK5*** | 6 | het | / | / | 1 |
| ***LAMB2*** | 3 | het | AR | Pierson syndrome | 2 |
| ***LYZ*** | 12 | het | AD | Amyloidosis, renal | 1 |
| ***MUC1*** | 1 | het | AD | Tubulointerstitial kidney disease | 1 |
| ***NIPBL*** | 5 | het | AD | Cornelia de Lange syndrome 1 | 1 |
| ***NOTCH2*** | 1 | het | AD | Alagille syndrome 2 | 1 |
| ***NPHS1*** | 19 | het | AR | CNS/SRNS | 5 |
| ***NPHS2*** | 1 | het | AR | CNS, SRNS | 5 |
| ***NPNT*** | 4 | het | / | / | 1 |
| ***NUP107*** | 12 | hom | AR | Childhood SRNS | 1 |
| ***OCRL*** | X | hemi | XR | Lowe syndrome | 1 |
| ***PAX2*** | 10 | het | AD | Adult-onset FSGS without extrarenal manifestations | 7 |
| ***PKD1*** | 16 | het | AD | Polycystic kidney disease 1 | 2 |
| ***PKHD1*** | 6 | het | AD | Polycystic kidney disease 4, with or without hepatic disease | 1 |
| ***PLCE1*** | 10 | het | AR | CNS/SRNS | 2 |
| ***SLC5A2*** | 16 | het | AR | Renal glucosuria | 1 |
| ***TBC1D8B*** | X | het | XD | Early-onset SRNS with FSGS | 1 |
| ***TNS1*** | 2 | het | / | / | 1 |
| ***TRIM8*** | 10 | het | AD | Focal segmental glomerulosclerosis and neurodevelopmental syndrome | 1 |
| ***TRNL1*** |  |  |  |  | 2 |
| ***TRPC6*** | 11 | het | AD | Familial and sporadic SRNS (mainly adult) | 3 |
| ***TTC21B*** | 2 | het | AD;AR | FSGS with tubulointerstitial involvement | 1 |
| ***UMOD*** | 16 | het | AD | Tubulointerstitial kidney disease | 1 |
| ***WT1*** | 11 | het | AD | Sporadic SRNS (children: may be associated with abnormal genitalia); | 13 |

*AD autosomal dominant, AR autosomal recessive, CNS congenital nephrotic syndrome, FSGS focal segmental glomerulosclerosis, SRNS steroid-resistant nephrotic syndrome.*
